# Remote Dynamic Three-Dimensional Scene Reconstruction

**DOI:** 10.1371/journal.pone.0055586

**Published:** 2013-05-07

**Authors:** You Yang, Qiong Liu, Rongrong Ji, Yue Gao

**Affiliations:** 1 Department of Electronics and Information Engineering, Huazhong University of Science and Technology, Wuhan, Hubei, China; 2 Department of Automation, Tsinghua University, Beijing, China; 3 Department of Cognitive Science, School of Information Science and Engineering, Xiamen University, Xiamen, Fujian, China; 4 School of Computing, National University of Singapore, Singapore; Institute of Psychology, Chinese Academy of Sciences, China

## Abstract

Remote dynamic three-dimensional (3D) scene reconstruction renders the motion structure of a 3D scene remotely by means of both the color video and the corresponding depth maps. It has shown a great potential for telepresence applications like remote monitoring and remote medical imaging. Under this circumstance, video-rate and high resolution are two crucial characteristics for building a good depth map, which however mutually contradict during the depth sensor capturing. Therefore, recent works prefer to only transmit the high-resolution color video to the terminal side, and subsequently the scene depth is reconstructed by estimating the motion vectors from the video, typically using the propagation based methods towards a video-rate depth reconstruction. However, in most of the remote transmission systems, only the compressed color video stream is available. As a result, color video restored from the streams has quality losses, and thus the extracted motion vectors are inaccurate for depth reconstruction. In this paper, we propose a precise and robust scheme for dynamic 3D scene reconstruction by using the compressed color video stream and their inaccurate motion vectors. Our method rectifies the inaccurate motion vectors by analyzing and compensating their quality losses, motion vector absence in spatial prediction, and dislocation in near-boundary region. This rectification ensures the depth maps can be compensated in both video-rate and high resolution at the terminal side towards reducing the system consumption on both the compression and transmission. Our experiments validate that the proposed scheme is robust for depth map and dynamic scene reconstruction on long propagation distance, even with high compression ratio, outperforming the benchmark approaches with at least 3.3950 dB quality gains for remote applications.

## Introduction

Depth maps are crucial for three-dimensional (3D) imaging and display, which have been widely deployed in 3D virtual scene perception [1], [2], 3D shape analysis [3], and 3D reconstructions of cells, objects [4]–[6], and organs [7]. In addition, with the development of distributed networks, remote monitoring and processing applications are emerging [8], for instance remote medical imaging [9], remote monitoring [10], and remote clinical routine [11]. To improve the remote medical processing accuracy, remote reconstruction of dynamic 3D scene with the combination of 3D image processing and video streaming will be helpful. In these systems, the dynamic scene rendering using color video and the corresponding depth maps can only be done at the terminal side, which therefore requires compressing and sending the color video and depth maps from the capturing side.

It is widely recognized that the depth maps with video-rate and high resolution synchronized to the color video are the precondition for high quality of reconstructed dynamic 3D scene [5], [12]–[16] However, the computation limitation at the capturing side restricts its quality of depth map reconstruction. For example, the widely used RGB-D [12], [17] (e.g., Kinect) and ToF [13], [18] cameras can only capture the depth map in video-rate with very low resolution, e.g., 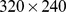 pixels, which are very difficult to achieve higher resolution, e.g., standard definition or even higher. Another issue raises from the extra bandwidth cost by sending the depth map beside the color video stream for telecommunication. Given both above restrictions, in most cases, only color video are compressed and transmitted to the terminal side for dynamic 2D scene presentation rather than 3D reconstruction.

Recent years have witnessed a large popularity to directly reconstruct the depth map and dynamic scene at the terminal side [19]. One representative work comes from the propagation based algorithms, which has been shown to be an efficient way to construct the continuous depth of a dynamic scene in the time-domain [20], [21]. In depth propagation, the variation of a scene is assumed to be identical for both the depth and color videos. The motion information, e.g. motion vectors (MVs), can be projected to depth maps among color frames. Specifically, objects keep static in consequent color frames will not arouse depth value variations, and the depth values for the region containing static object maintain static in the depth map. On the other hand, motion in consequent color frames correspond to depth value variation in the same region. Therefore, the status (i.e., static or moved) of objects in consequent color frames can be used to describe the depth value variation in the depth map. The MVs have been widely applied to describe the motion status of objects, and thus the accurate MVs are highly required for the precise reconstruction of remote dynamic 3D scene. The existing works on propagation based algorithm mainly focus on improving the color video capturing on the encoder side [20], [22]. Given the high-quality color videos as in [20], [22], accurate MVs computed via optical flow or motion estimation can be expected. Such accurate MVs can then be applied in propagation for depth reconstruction.

**Figure 1:**
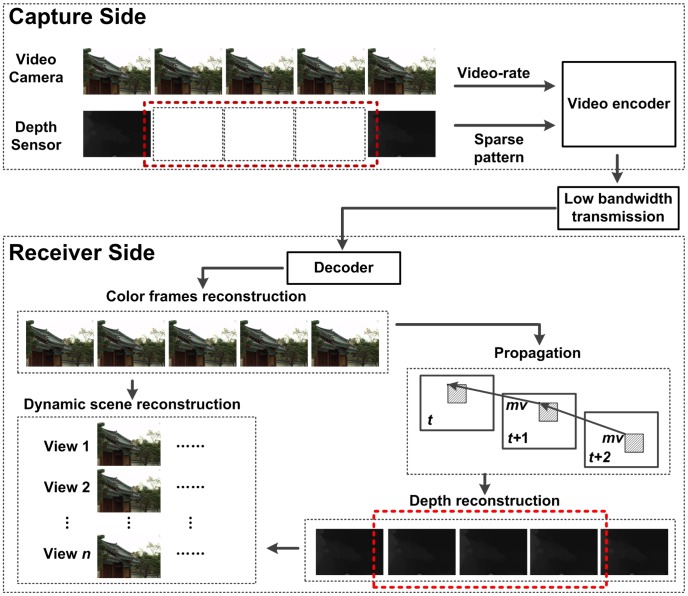
A systematic overview of the remote dynamic scene reconstruction system.

However, as shown in Figure 1, for the case of capturing scene remotely and dynamically for depth reconstruction, the color video has to be compressed at the encoder side, and therefore the decoding at the terminal side has to face dramatic compression loss. If directly applying the existing propagation based methods on the decoded color video, the accuracy of depth propagation and reconstruction is doom to be significantly degenerated because of the large compression ratio, especially when the transmission bandwidth is limited. Therefore, it is a fundamental problem to propagate the depth of the dynamic scene from the reconstructed color video bitstream to reinforce each other. However, such an emerging topic retains unexploited so far.

Actually, accurate MVs are available in the compressed video stream, for which the MVs are calculated based on the original color video without any compression losses [23], [24]. That said, MVs are the basic information for video coding technique to squeeze out the temporal redundancy. These vectors can be encoded and transmitted to the terminal side for color video decoding. Motivated by this, we propose a scheme for remote dynamic 3D scene reconstruction by means of propagation. In this scheme, a remote reconstruction scheme for dynamic 3D scene at the terminal side, avoiding in-accurate MV calculation from the highly compressed color videos. In order to process the MV properly, we rectify MVs from compressed color video stream, where MVs are calculated for rate-distortion minimization rather than the purpose of propagation. After that, a serial of spatial processing methods to improve the quality of the reconstructed depth map, including multi-directional spatial prediction for propagation hole due to absent MVs, and a boundary enhancement to avoid the foreground and background cross-talk effect due to block-wise MVs around object boundary. For the terminal device, the proposed scheme can remotely reconstruct the dynamic 3D scene with high quality, low communication bandwidth, and low computation resource requirement.

## Materials and Methods

### Problem Description

In this section, the compression techniques for video coding are analyzed to be used for both the depth propagation and the dynamic 3D scene reconstruction. Serving as a protocol between the encoder and the decoder, the compression information includes the descriptors for prediction modes (i.e., Inter or Intra modes), MVs, encoding modes (i.e., prediction direction for Intra mode), and block modes (i.e., block size of 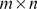). The compression information is used by the decoder to reconstruct all the compressed blocks in an image or a frame, in which the block is the basic processing unit of video coding. Let 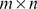 be the block size, ranging from 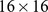, 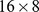, 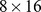, 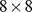, 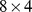, 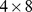, to 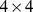. According to the MPEG compression standard, all the compression information is calculated and determined at the encoder side based on the original high quality color videos. For a block encoded by Inter mode, the best matched block in the reference frame is determined by motion estimation, from which MV is determined by their coordinate differences. On the other hand, for one block encoded by Intra mode, the color information inside this block is predicted by the up-left, up, up-right and left neighbors in the current frame rather than the reference frame. Usually, a block can be encoded by Inter mode if it contains few textures or even without texture, while being encoded by both the Intra and Inter modes when the block crosses the object boundary.

As mentioned above, there is an assumption for depth propagation: the motion information is identical for both color and depth video [20], [22]. This makes sense in the previous works, where the motion information is depicted by pixel-wise MV that propagates the depth value pixel-by-pixel from the previous frame to the current one. However, as for the remote dynamic scene reconstruction, only compression information in video stream can be used at the terminal side. Therefore, the depth map reconstruction is totally different from the traditional method. For example, the obtainable MV is block-wise rather than pixel-wise, and the reconstruction method should be designed according to each of the specific prediction types. Under such a circumstance, several problems may occur when applying the compression information to the depth reconstruction, including:

1. *MV Outliers.* Motion field of a dynamic scene is a Markov field in both the spatial and the temporal. According to this property, for a block inside an object, the depth for all its sub-blocks should keep consistent among each other. However, the MV for each block is determined independently by rate distortion optimization [23], [24], without considering the consistency with neighbors. This inconsistency can easily lead to MV outliers, which will result in unsmooth depth propagation and reconstruction. Thus, during reconstructing a given block, the outliers should be detected and processed properly to avoid imprecise depth values.
2. *Absent MVs.* As mentioned above, a block may be encoded by Intra mode without MV for depth propagation. In this case, there will be a propagation hole for this block since no depth value can be propagated from previous frame.
3. *Near-boundary blocks.* The boundary of a moving object divides a region into foreground and background, Both parts have different MVs. When a block covers the boundary, the MV will assign the same vector value to all pixels in this block without recognition of the difference between the foreground and the background. This assignment will break the boundary and result in dislocations in depth reconstruction.

### Overview of the Proposed Scheme

**Figure 2:**
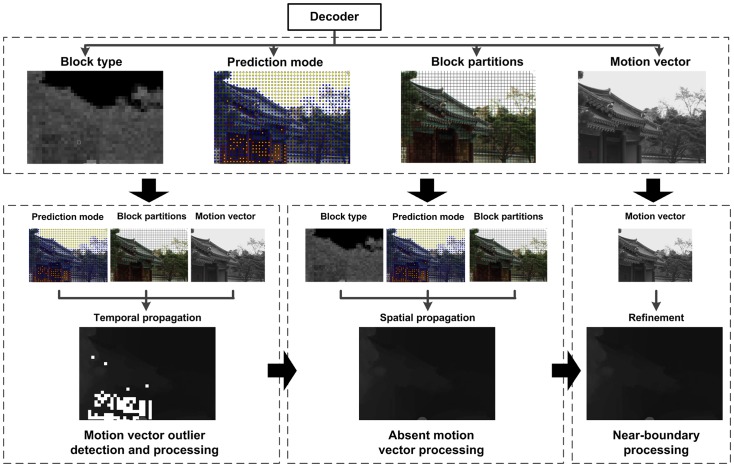
Framework of our proposed scheme for video-rate depth reconstruction via compression information in compressed color video bitstream.

In this work, we propose a scheme for remote reconstruction of dynamic 3D scenes based on the compression information in the compressed color video stream. The flowchart of the proposed scheme is shown in Figure 2. In this scheme, the compression information, including the prediction mode and MVs, is extracted from the color video stream. Then, the MV outliers will be detected and rectified to assure the obtained MVs are correct for the temporal propagation based depth reconstruction. After that, for the Intra block without MVs, the spatial propagation which utilizes the Intra mode information is proposed to generate the absent MVs. Furthermore, to improve the quality of dynamic scene reconstruction, a specific algorithm is designed for boundary enhancement on depth map.

### MV Outlier Elimination

As mentioned above, the block modes and MVs for all blocks are crucial for reconstructing the depth of a given block at the decoder side. The block mode corresponds to the block size 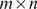, and the MV 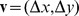 is a tuple identifies the location for the best matched block with size 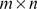 in the reference frame. More specially, the depth reconstruction in terms of propagation for current block 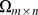 can be expressed as
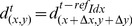
(1)where 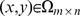 is the coordinate for one pixel in the current block 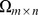 of the depth frame at time 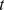, 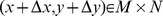 locates the correspondent coordinate for the pixel at 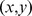 in the depth frame 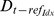 with size 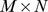 at the time 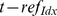, where 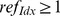 is the time stamp for the reference frame. By using Equation (1), a block of depth 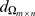
in the depth frame 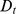 can be easily reconstructed via depth propagation by the coordinate 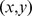. And this is done in an ergodic manner if all of the correspondent pixels can be located by vector 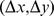 in the depth reference frame 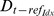.

As described previously, the MV of a block should keep consistent when comparing to the adjacent neighboring vectors, especially when these MVs describe the movement of one object. Furthermore, the object boundary divides a region into both foreground and background parts. And the MVs for these two parts are different. Therefore, we should locate the block before determining whether the MV of this block is an outlier.

**Figure 3:**
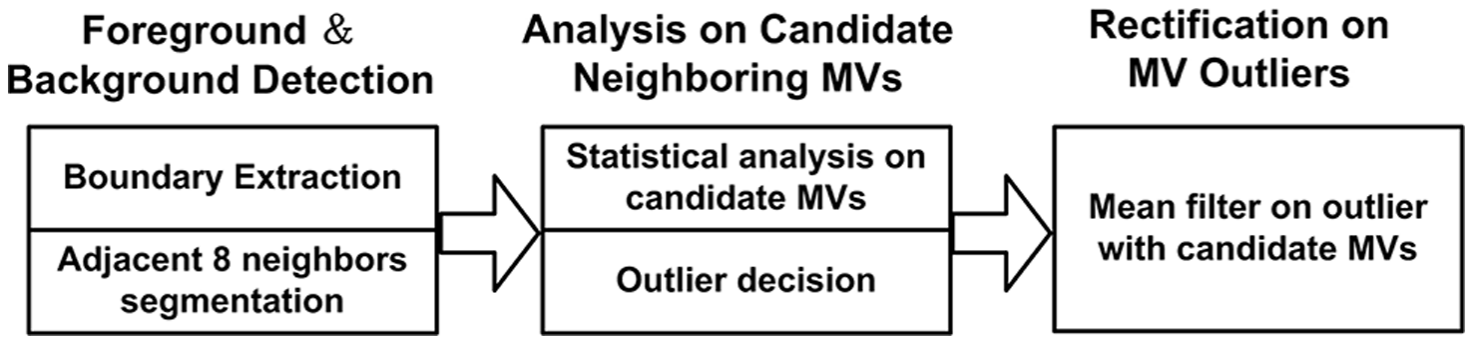
Flow chart for MV outlier detection and processing in our proposed scheme.

To this end, we propose a scheme shown in Figure 3 for the MV processing. In this scheme, all MVs of blocks are processed by 3 steps to eliminate the outliers. In the first step, a foreground and background detector is applied on the block to locate which part this block belongs to. The object boundary is crucial in distinguishing foreground and background, and thus a texture extractor 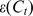 can be applied to generate a texture mask map 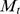 on this color image. Let 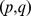 be the coordinate of current block 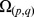 in 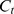, where 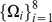 are the 8 adjacent neighboring blocks around 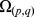, 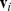 is the corresponding MV for 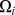, 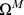 and 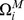 is the corresponding block for 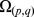 and 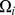 in 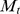 respectively, where 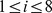. There are three cases for foreground and background detection:

- For the case that texture appears in 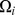, foreground and background can be distinguished by MVs. No matter which part the current block belongs to, the neighboring blocks belonging to the other part will not be involved for latter outlier decision.
- For a block crossing the boundary of foreground and background, this is one of the problems mentioned above and this block will be processed latter in Section 3.3.
- Finally, there is no foreground and background parts if there is no texture appears in both 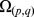 and 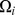. In this case, all MVs of neighboring blocks will be involved for outlier detection.

After the involved neighboring MVs have been determined, we can calculate the statistical properties, including average and deviation, of these MVs for outlier detection. The MV of current block is outlier if it is quite different by means of these properties. We summarized the above analysis into Table 1 for foreground and background detection.

**Table 1:** Algorithm 1: Foreground and Background Extraction.

**Step 1**. Use 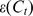 to obtain 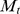 .
**Step 2**. If 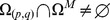 , mark 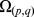 as texture block and terminate.
**Step 3**. Remove 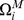 from adjacent neighboring block set if 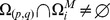 , 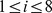 , and obtain
candidate set 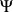 .
**Step 4**. Use 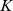 -mean clustering algorithm on 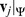 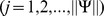 to group MVs of 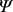 into set 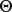 .
**Step 5**. If 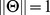 , we have MV candidate set 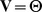 .
Else 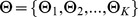 , and we have
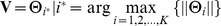
**Step 6**. Calculate average 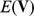 and standard deviation 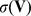 of 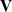 , 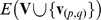 and standard deviation 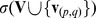 of 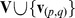 . Calculate
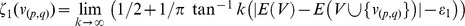 (4)
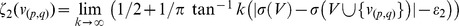 (5)
where 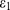 and 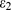 are threshold average and standard deviation respectively. 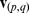 is outlier when
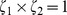 .

Equations 2 and 3 are in form of the Heaviside step function. The function is a unit step function, and it can be denoted by:
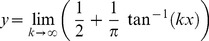
(2)


**Figure 4:**
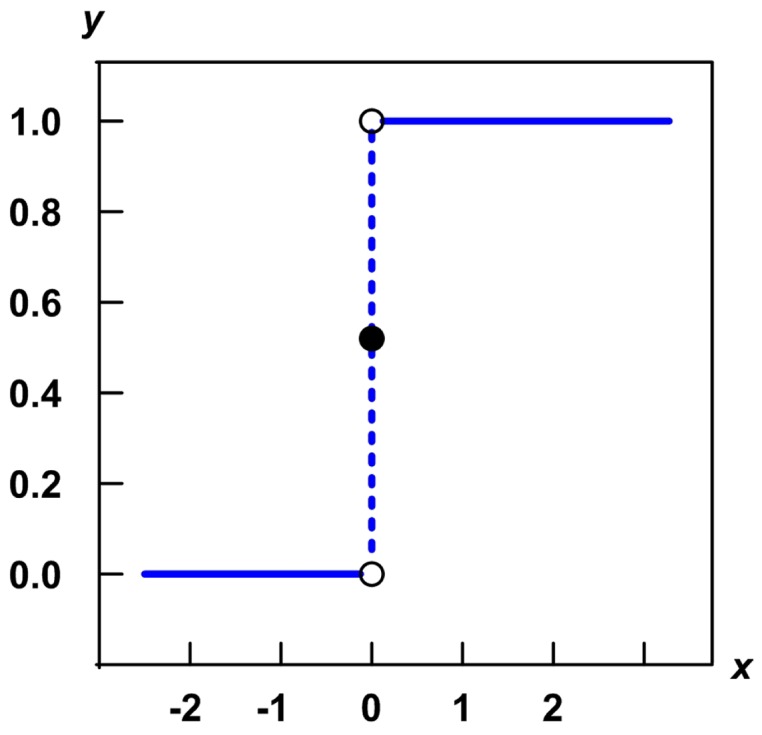
Heaviside step function.

This function is always applied in the mathematics of control theory and signal processing. This function is a discontinuous function whose value is 0 for negative argument and 1 for positive argument. As shown by Figure 4, the function represents a signal that switches on at a specified time, as usually triggered by a threshold, while staysing switched on indefinitely.

After applying this algorithm, we use a mean filter to MV if this MV is determined as an outlier.

### Absent MV Processing

The block encoded with intra mode has no MVs for propagation and reconstruction. In this case, the depth information of the current block is reconstructed spatially by the depth from adjacent blocks.

**Figure 5:**
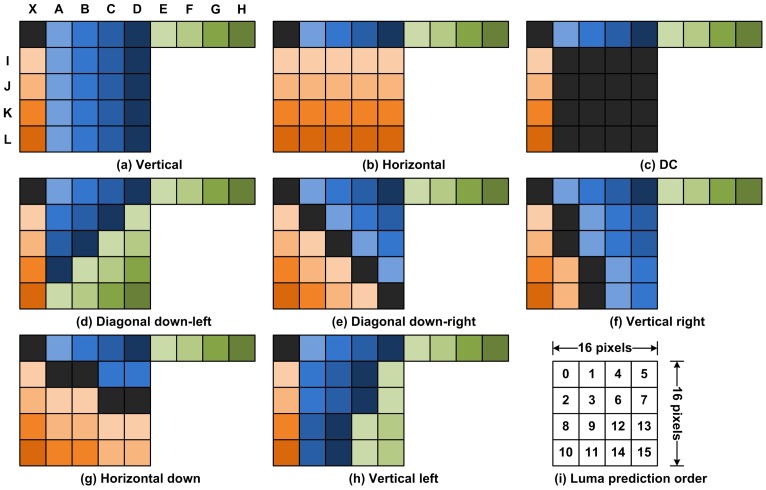
Block order of macroblock and Intra predictions for one 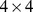 block.

According to the Intra prediction techniques adopted in the MPEG video coding standards, a block can be predicted spatially by its neighboring pixels [23], [24]. For example, Figure 5(a–h) shows a 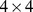 block that can be spatially predicted by its surrounding pixels 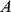, 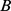, 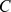, 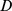, 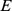, 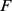, 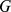, 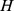, 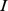, 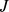, 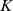, 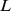 and 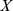. And the coding mode includes vertical, horizon, DC, down left, down right, vertical left, vertical right, horizon down and horizon up. For example, when the block is encoded by the DC mode, the color information of all pixels can be calculated as
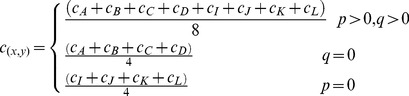
(3)


As for a color video frame, one block with size 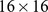, 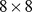 or 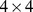 coded by Intra mode can be reconstructed by the color information of its available neighboring pixels. The best Intra mode is chosen for every block due to its spatial features, which also can be used to depth map reconstruction.

Similar to the reconstruction on color information, spatial depth reconstruction for one block can be also performed in the same manner.

### Near-boundary Depth Processing

We define a current block 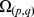 as a near-boundary block when 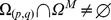. As mentioned previously, object boundary is a division for foreground and background parts with different MVs. The pixels inside 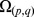 should be processed separately. However, the current block 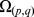 has only one MV 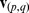 for all pixels in the procedures of depth propagation and reconstruction. The reconstruction will break the object boundary and result in dislocations. Usually, depth map with artifacts on boundaries results in a failure on foreground-background separation [25]. In order to solve the dislocation problem, one solution is to predict a pixel-wise MV based on available block-wise MVs for each of the pixels in 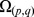.

In video coding standards like MPEG, overlapped block motion compensation (OBMC) was a good solution that not only improves the prediction accuracy but also avoides the blocking artifacts [26]. When using OBMC, blocks are typically twice bigger in each dimension and are overlapped quadrant-wise with all 8 neighboring blocks. Thereafter, each pixel belongs to 4 blocks. In this scheme, there are 4 predictions for each pixel, which are summed up to a weighted mean. For this purpose, blocks are associated with a window function that has the property that the sum of 4 overlapped windows is equal to 1.

**Figure 6:**
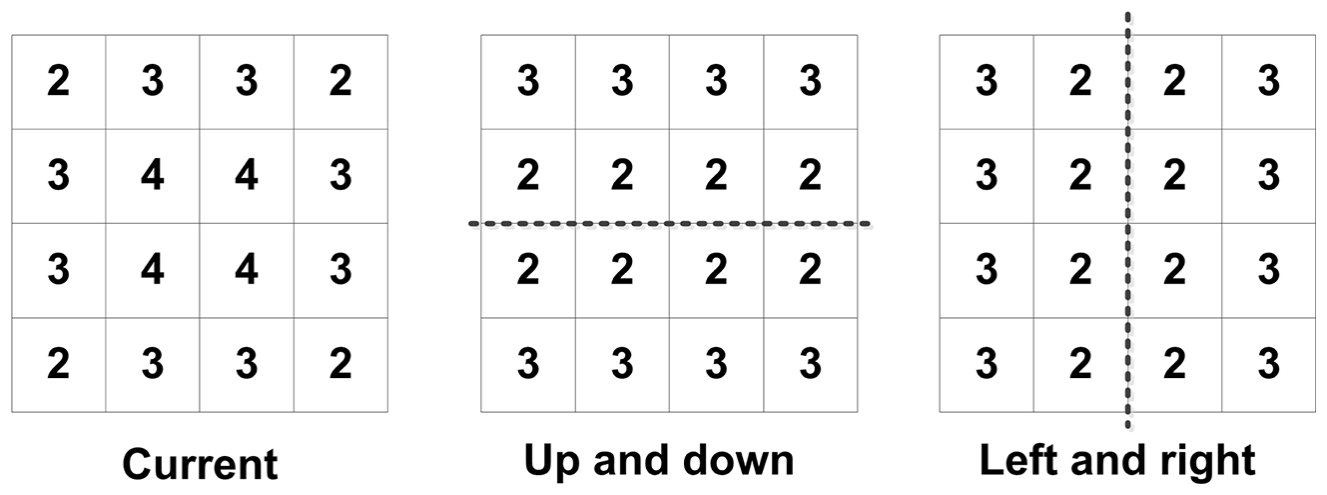
The window function corresponding to the weighting coefficients.

Figure 6 shows an example of weight coefficient windows for OBMC. In this example, to predict the pixel on the top left corner of the block, the weights associated with the MVs of the current, the top, and the left block are 2/8, 3/8, and 3/8, respectively. For the pixel on the first row and the second column, the weights are 3/8, 3/8, and 2/8 respectively. While improving the prediction accuracy and avoids the blocking artifacts, OBMC cannot be applied in decoder when MV is determined. Meanwhile, OBMC has not been exploited in depth reconstruction in previous researches.

There are two problems which may occur when applying OBMC for depth reconstruction. First, MVs from neighboring blocks may be not available in decoder when the whole macroblock or several blocks in macroblock are Intra encoded. Originally, in traditional OBMC, the MVs can be predicted by neighboring blocks, or calculated again by motion estimation precisely since the original color frame is available. However, in the perspective of depth reconstruction at terminal side, the neighboring block may be Intra coded without MV for prediction, while no original color frame being available for accurate motion estimation. Second, when the whole macroblock is Inter encoded, MVs for different blocks may point to different direction with different norm. In this case, it is difficult to determine which MV can be involved for prediction. In this case, neighboring MVs for prediction and the weight coefficient window should be modified accordingly.

In order to solve the above problem, we improve OBMC adopted in H.264 [23], [24] by proposing a modified OBMC (MOBMC). In MOBMC, a block-wise step motion estimation method on reconstructed color image to determine the neighboring MVs for prediction. In the procedure of motion estimation, the block search is performed in block-wise step, i.e., if the current candidate block is on 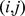, the next candidate block is then on 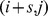 or 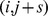, where 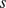 is the block size which is a constant. We can find that, after the current color frame has been decoded, at most 4 blocks that Inter encoded in up, down, left and right neighboring macroblock separately match best to the current block 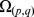. The contents of the best matched block should be similar or exactly the same with the current block. Therefore, a similarity metric 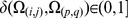 proposed in [27] is calculated in matching process. The two blocks are identical when the metric 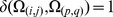. Based on this metric, the block 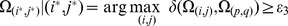 will be selected as the candidate block for prediction.

After all the candidate blocks have been determined, a prediction of MV for each pixel in the current block can be performed based on weight coefficient windows. Traditionally, the weight coefficient windows in OBMC assign more weights on the current block [24], which is beneficial from high accuracy motion estimation in encoder once the original color frame is available. However, when accurate motion estimation is unavailable, more weights for adjustment should be given to neighboring blocks. Therefore, we further modify the traditional weight coefficient window for OBMC for depth reconstruction. In our modification, the coefficients are empirically set and given in Figure 6. In this window, more weights are given to MVs from neighboring blocks.

### Dynamic 3D Scenes Materials

Dynamic 3D scenes contain a video-rate color image sequence that records the motion, color and texture information of this scene. Furthermore, a high-resolution depth map sequence is also captured to record the 3D space information for all visible objects. Note that this high-resolution depth map cannot be captured by RGB-D or ToF cameras in video-rate, as being synchronically with the color image sequence so far. Recently, MPEG released their standard test sequences for dynamic 3D scenes with high resolution (more than standard definition) and high frame-rate, including color images and depth maps [28]. The color images were captured by cameras, but the depth maps were not captured but calculated by stereo matching and even manual labeling. The quality of the depth maps obtained through this way were assumed with the best quality to be obtained.

The dynamic 3D scene materials named as *Undo Dancer*, *Lovebird1*, and *Kendo* will be used to testify our proposed algorithm. The captured color images and the calculated depth maps of these materials are selected from [28]. These materials are with different challenges in depth reconstruction and dynamic 3D scene reconstruction, as listed in Table 2. It is worth to note that these materials are standard test materials provided and recommended by MPEG, and are widely used in experiments of dynamic 3D scenes reconstruction.

**Table 2:** Challenges of test sequences.

Test Sequences	Challenge
Dancer	Full tiny but orderless textures: miss-matches in motion estimation.
Kendo	Illumination variation and focus-light rotation: non-zero MV for static region.
Lovebird1	Zooming out movement: overlap of neighboring MVs.

## Results and Discussion

### Experiment Settings

Since the compression ratio is highly related to the quality of depth reconstruction. we first reconstruct depths via color video bitstream with different compression ratios. Then, we compare: 1. their rate-distortion gains are compared, 2. the bit rate savings on the same reconstruction quality range (measured by BDBR [29]), and 3. the subjective quality of scene reconstruction between the proposed scheme and the benchmarks. The parameters BDBR and BDPSNR show the average performance gain between two comparison data sets under the same condition.

Our experiment is carried out on standard test sequences, including *Dancer*, *Kendo* and *Lovebird1* from the MPEG 3DV work group [28]. Table 2 provides the challenge descriptions for these sequences. In order to verify the quality of depth reconstruction, we apply the reconstructed depth maps for virtual view synthesis in the remote dynamic scene reconstruction. The standard platform VSRS [28] for virtual view synthesis is adopted for scene reconstruction.

Table 3 lists our experiment setting, in which color and depth frames are encoded by the same coding settings, including the prediction structure and the quantization parameter (QP). We set the color video and periodic key depth maps as available, and evaluate the quality of the reconstructed depth maps using the ground truth depth maps provided by MPEG 3DV work group [28]. The key frame period for depth reconstruction is set as 8, indicating that there are 7 depth maps need to be reconstructed between each pair of key depth frames. The key frame period is a typical setting for remote video transmission system. In this case, the number of encoded depth frames is 1/8 for color frames, and this can considerably save transmission bandwidth and therefore benefit the remote scene reconstruction. As the comparison benchmark, we encode all the depth maps with the same prediction structure of color videos, and find the quality performance under the same bit-rate of these two schemes. On the other hand, MOBMC in our scheme can acheive better perceptual quality around object boundaries. To prove this statement, we also compare the depth map that reconstructed by direct propagation without MOBMC.

**Table 3:** Experiment settings and coding parameters.

Item	Settings
Prediction Structure	IPPP
Number of reference	1
Quantization parameter	22, 32, 37, 42 (Kendo, Lovebird1) 22, 32, 44, 48 (Dancer)

In our experiments, we empirically set the thresholds 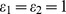 and 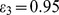, and the 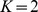. The thresholds 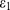 and 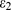 are tolerance values for MV outlier detection, and we will discuss them in the next subsection. As for 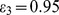, it is designed for the similarity metric 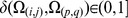. In practice, the two blocks are almost the same when 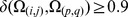 [27]. On the other hand, the parameter 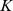 for clustering is set as 2, since only 2 groups are needed to distinguish the foreground and background.

### On the Parameter 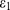 and 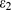


From Equations 2 and 3 we can see that there are two thresholds 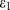 and 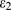 in our formulation. Both modulate the number of the motion vector outliers, and thus the final quality of the reconstructed depth maps. These parameters (i.e., thresholds) are usually used in the motion vector outlier classification. For parameter 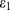, it is a real number in the interval 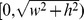, where 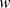 and 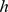 are the width and height of image respectively measured by pixels. This parameter determines the uniform degree of object motion in a certain range of blocks. If 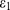 tends to be small, the motion degree of a certain range of blocks is small, and 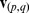 can be easily figured out with even tiny difference from other vectors. Therefore, 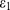 is usually set bigger than 0 in order to avoid over estimation on the outliers. On the other hand, 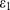 is set as an integer number rather than a real number in the case of video coding, since MV is calculated by comparing pixel positions, therefore only integer MV can be obtained. As for parameter 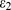, it is a real number for the eccentricity of a set of MVs. 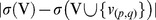 in Equation 3 indicates the difference of 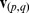 when comparing to its surrounding MVs. Therefore, 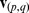 can be hardly treated as outlier if 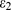 is higher.

**Figure 7:**
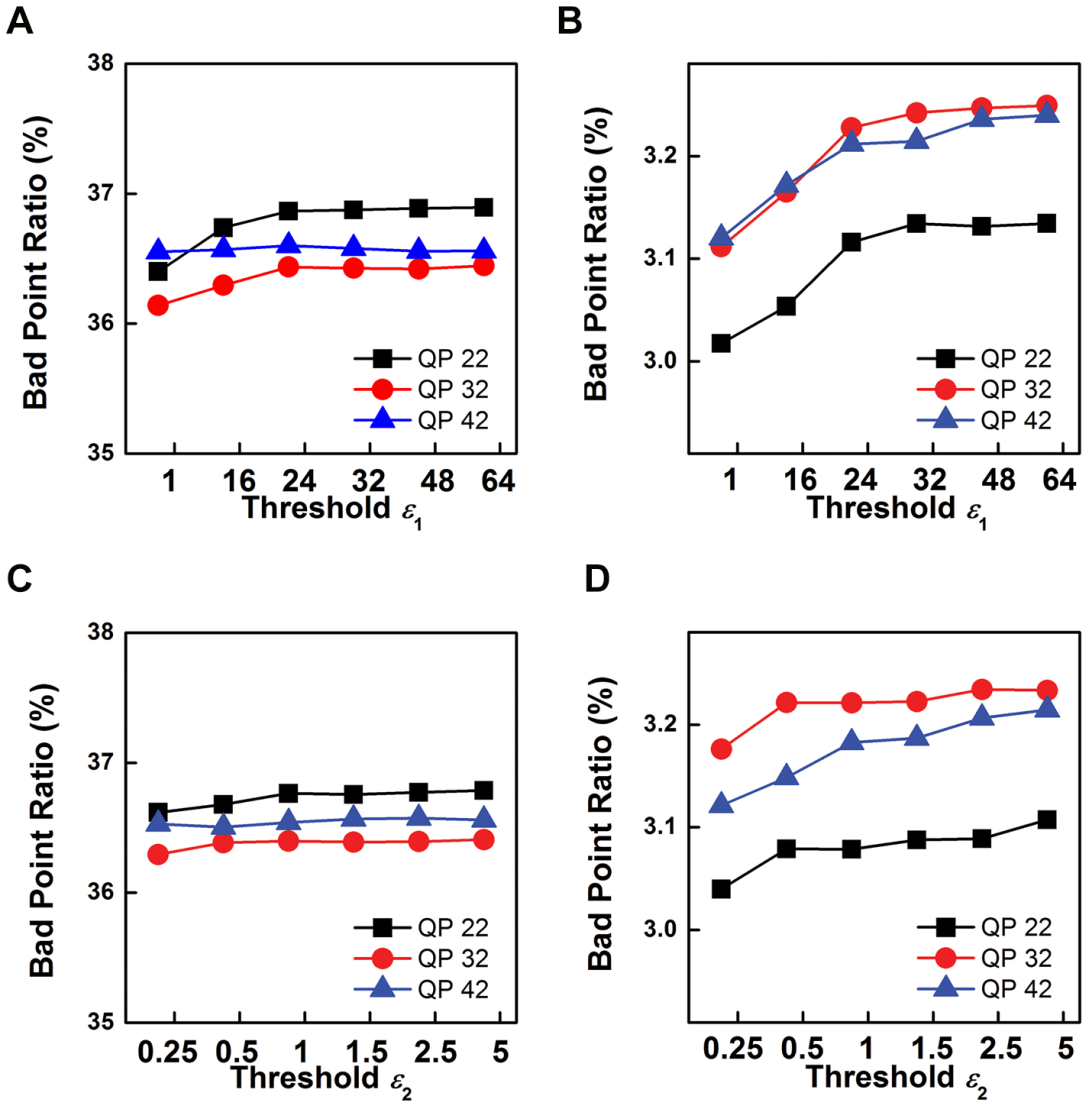
Parameter analysis for thresholds 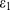 and 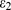 on the Kendo and Lovebird test materials in the case of using different QP parameters. (A) Bad Point Ratio curves for different 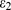 and QP parameter when 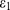 is fixed as 8. Experiments are performed on Kendo test sequence. (B) Bad Point Ratio curves for different 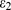 and QP parameter when 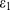 is fixed as 8. Experiments are performed on Lovebird test sequence. (C) Bad Point Ratio curves for different 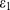 and QP parameters when 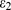 is fixed as 1. Experiments are performed on Kendo test sequence. (D) Bad Point Ratio curves for different 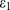 and QP parameters when 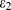 is fixed as 1. Experiments are performed on Lovebird test sequence.

Figure 7 demonstrates the analysis on 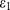 and 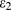 when different test materials (Kendo and Lovebird) and QP settings (22, 32 and 42) are involved. Bad Point Ratio is employed to measure the quality of reconstructed depth map, and a pixel is defined as bad point if the the corresponding depth value difference between reconstructed and benchmark depth maps is bigger than 1. The Bad Point Ratio in Figure 7 is an average value that is calculated by 16 sequentially reconstructed depth maps. Figures 7 (A) and (B) show that the variation curves of 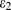 when 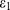 is fixed as 8. These curves indicate that for both the test materials and all QP settings, the Bad Point Ratio becomes bigger and tends to be stable when 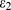 becomes bigger. Practically, smaller Bad Point Ratio is preferred for depth map reconstruction. However, the curves are not stable when 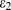 is small, and thus smaller 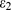 may be unsuitable for most test materials. As shown by Figures 7 (A) and (B), the performance of all curves tends to be stable when 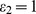, and the Bad Point Ratio is small enough for practical usage. Therefore, we set 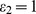 in the rest of our experiments. After that, 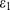 is analyzed based on the above discussion of 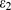, and the results are given by Figures 7 (C) and (D). These curves also indicate that for both test materials and all QP settings, the Bad Point Ratio becomes bigger when 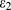 is become bigger. All curves have similar trend that smaller Bad Point Ratio can be obtained by smaller 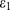. Therefore, the smallest 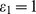 is selected.

Based on the analysis in Figure 7, the parameter settings for Equations 2 and 3 is settled with 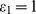 and 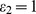.

### Results of Depth Map Reconstruction

**Figure 8:**
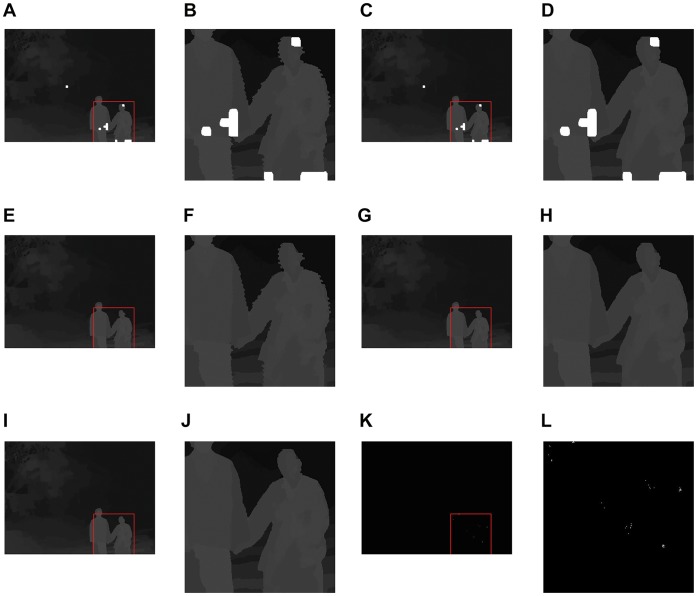
The results of each step in our scheme to the depth map reconstruction. (A) Depth map reconstructed by Equation 1. (B) The enlarged part in (A) for comparison. (C) Depth map reconstructed with MV outlier elimination from our scheme. (D) The enlarged part in (C) for comparison. (E) Depth map reconstructed with absent MV processing but without MV outlier elimination from our scheme. (F) The enlarged part in (E) for comparison. (G) Depth map reconstructed with MV outlier elimination and absent MV processing but without near-boundary depth processing from our scheme. (H) The enlarged part in (G) for comparison. (I) Depth map reconstructed with our scheme. (J) The enlarged part in (I) for comparison. (K) The difference map between (G) and (I) after near-boundary depth processing is applied. (L) The enlarged part in (K) for comparison.

We first show the effectiveness of each step in our scheme to the procedure of depth map reconstruction by Figure 8.

Our scheme is a combination of processing steps based on Equation 1, including MV outlier elimination, absent MV processing and near-boundary depth processing. As described above, each step is designed to solve one problem in the procedure of depth map reconstruction. Figure 8 demonstrates a reconstruction procedure on the Lovebird test material, which clarifies the problems during the reconstruction procedure, as well as how our scheme handles them. First, after all MVs are extracted from compressed video bitstream, depth map is mainly reconstructed by Equation 1, and the result is shown by Figure 7(A). In this figure, we can find that most of the depth information is reconstructed, since most of the blocks in this depth video are encoded as Inter mode. Some holes (i.e. blocks) in white appear in the reconstructed depth map, because these holes are encoded in the Intra mode, and MV is absent for reconstruction. Second, dislocations on object boundaries are obvious and they will cause faults in later 3D scene reconstruction. The reason for these dislocations is the MV outlier, which should be detected and processed properly. Therefore, we use the method of MV outlier elimination on Figure 7(A), producing the result in Figure 7(C). It can be found that the dislocations around object boundaries disappeares, and therefore the quality of depth reconstruction is improved. Third, the holes in white remain and need to be filled by the method of absent MV processing. Figure 7(E) shows the result of Figure 7(A) being processed directly by absent MV processing without the MV outlier elimination. The holes inside disappear but the dislocations on object boundaries remain unchanged. Therefore, we can know that the methods of MV outlier elimination and absent MV processing are important to reconstruct a complete depth map. Thus, we first use MV outlier elimination then absent MV processing on Figure 7(A) to obtain Figure 7(G). In this result, the depth map is completely reconstructed without holes and dislocations on object boundaries. It should be noted that absent MV processing cannot be performed before Equation 1 and MV outlier elimination. According to the method described by Figure 5, the depth value can only be predicted after the surrounding depth values are properly set. After Figure 7(G) is obtained, the method of near-boundary depth processing is applied on it to improve the quality of reconstruction, especially on object boundary, and the result is Figure 7(I). This processing is necessary to to avoid the failure on foreground-background separation [25]. From Figure 7(K), we can find that some depth values on object boundaries are adjusted, and the quality of reconstructed depth map is also slightly improved.

In summary, the proposed scheme is consisted of Equation 1, MV outlier elimination, absent MV processing and near-boundary depth processing. They are used sequentially and the order cannot be changed. Equation 1 can reconstruct most of the depth values, leaving with holes and dislocations. Subsequently, the MV outlier elimination is used to solve the problem of dislocation, and the absent MV processing is for holes. Finally, near-boundary depth processing is used for boundary enhancement and quality improvement.

**Figure 9:**
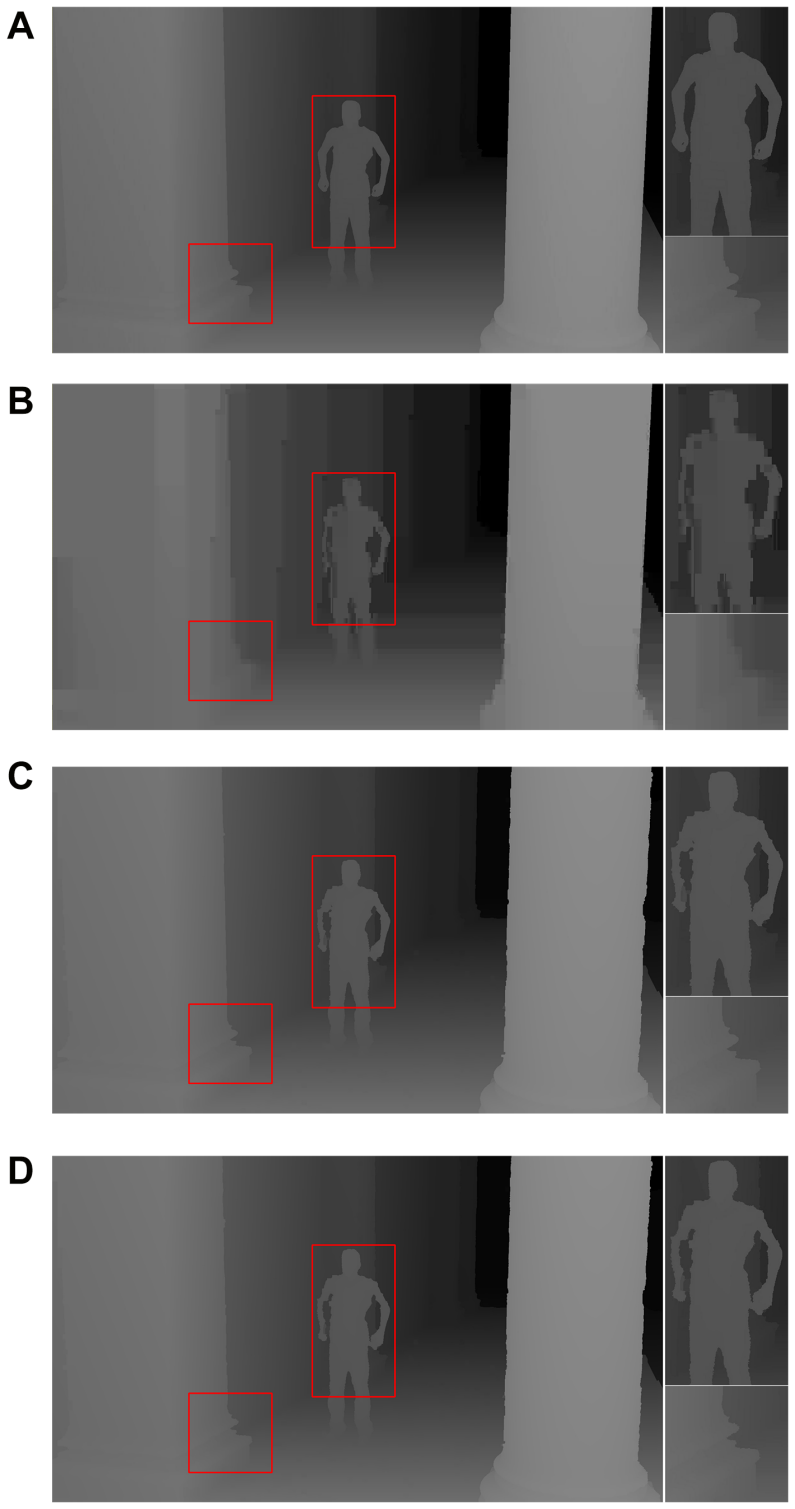
Comparisons on depth maps among the original, decoded, propagated and the proposed. The selected time-stamp is 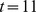. (A) Original depth map. (B) Decoded depth map (QP = 48). (C) Reconstructed depth map without near-boundary depth processing (QP = 48). (D) Reconstructed depth map with near-boundary depth processing (QP = 48).

After the single depth map reconstruction in Figure 8, we perform the experiments on consequent depth maps reconstruction, and the results are given by Figure 9. In this experiment, the selected time-stamp for depth map reconstruction is 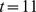. In this case, it can be known that there are 10 depth maps encoded as reference frames for the decoded frame as in Figure 9(B). On the other hand, the reconstructions of Figures 9(C) and (D) use the reconstructed depth map at 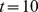, and consequently, the reconstruction of depth map at 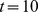 uses the map at 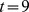, etc. The key depth map is at 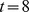, which is compressed by taking the depth map at 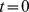 as reference. Besides that, the QP for Figures 9(B), (C) and (D) is set as 48, which is a very high parameter setting for low bandwidth communication.

The results in Figure 9 show the performance of our method. In case of low bandwidth communication (e.g. mobile communication), long distance prediction and high QP settings are benefit for bandwidth savings, but high quality losses are the byproduct of the settings. In our scheme, the prediction distance is trimmed and depth information is fetched from high quality key depth map. Besides that, MOBMC method is helpful to improve the quality of reconstructed depth map when high QP is selected. These methods in our scheme are useful for reconstruction of depth map with high quality even in low bandwidth communication cases. As shown by Figure 9(B), quality losses and blocky effects make the decoded depth map nearly unacceptable for latter scene reconstruction. The quality losses and blocky effects are caused by high QP and long distance prediction (the distance is 10 in this example) in compression procedure, and they are inevitable in low bandwidth communication applications. However, most of the information in the original depth map is reserved by our method, as shown by Figures 9(C) and (D). The performance is benefit from the short prediction distance, accurate MV in color video stream and MOBMC. On the other hand, Figures 9(C) and (D) show the robustness of our scheme, and the performance keeps stable even for longer distance reconstruction. Finally, the proposed MOBMC is beneficial for boundary enhancement and detail preservation. As depicted by the enlarged parts of Figures 9(A), (C) and (D), the detail information around the hands of the actor are reserved by the proposed MOBMC. Therefore, the experimental results show that our scheme is very useful in depth map reconstruction, even in low-bandwidth communication as well as long-distance propagation.

### Results of 3D Scene Reconstruction

**Figure 10:**
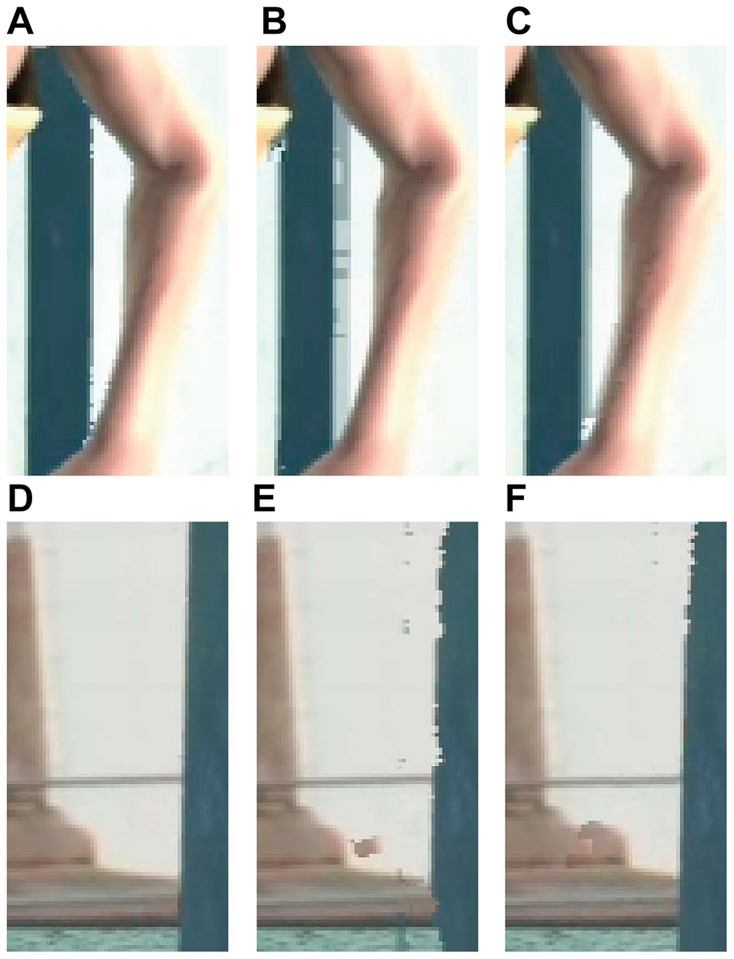
Subjective quality comparison for the proposed scheme and the benchmark. The images are synthesized by corresponding depth maps. (A) Decoded. (B) DP. (C) The proposed. (D) Decoded. (E) DP. (F) The proposed.

Based on the reconstructed depth maps, the dynamic 3D scenes can be built. The subject comparisons are given in Figure 10. In this figure, enlarged details are shown, and the details are synthesized by the decoded depth, the reconstructed depth with “DP” and the proposed scheme respectively. The scene synthesized by the decoded depth is served as quality baseline in the comparison. The scene synthesized by depth that reconstructed by “DP” contains distortions around boundaries, and the proposed scheme with MOBMC eliminates most of the distortions and improves the subjective quality, and the results are given in Figure 10.

### Results of Streaming Bandwidth and Quality

As shown by the above experimental results, our scheme has better performances on depth map and dynamic scene reconstruction. Based on the results, our scheme has the potential to be applied in remote medical processing and monitoring. In this case, the performance on streaming bandwidth and quality is important for remote applications.

**Figure 11:**
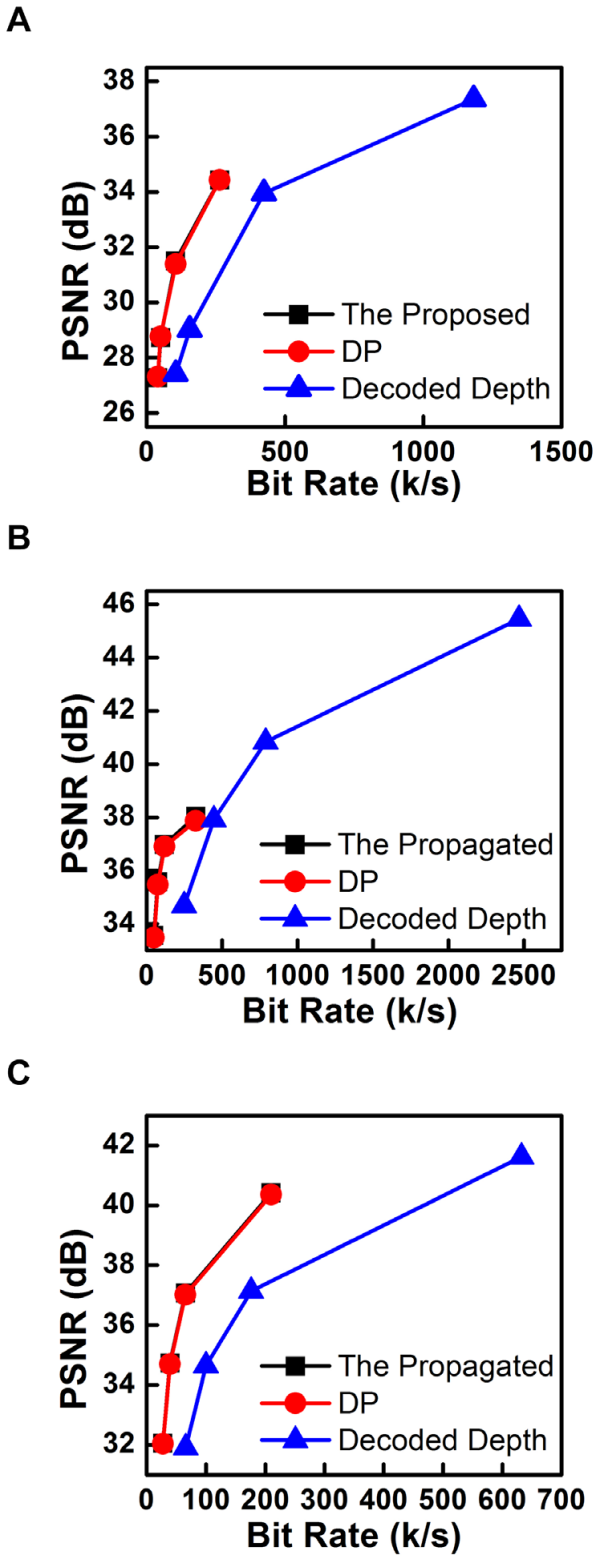
Rate distortion curves for the proposed scheme and the benchmarks. (A) Dancer. (B) Kendo. (C) Lovebird1.

The results of streaming bandwidth and quality are given by Figure 11 and Table 4, and obvious quality performance gains can be found for the proposed scheme on all test sequences. As for the rate-distortion performance comparisons, the propagation based depth reconstruction scheme is better than the benchmark, where all depth maps are available at the terminal side. The scheme label “DP” is for depth reconstruction by direct propagation without the MOBMC. As depicted in Figure 11, the rate-distortion curve for depth reconstruction is on the left and up side of the benchmark, indicating better rate-distortion performance than the benchmark. For further compare the bit rate savings on the same reconstruction quality range and the quality gains on the same bit rate range, the parameters of BDPSNR and BDBR are calculated, and the results are listed in Table 4.

**Table 4:** BDPSNR and BDBR performance comparisons.

Test	Scheme	BD range	BDPSNR	PSNR range	BDBR
Sequences		(kbps)	(dB)	(dB)	(kbps)
Dancer	DP	[39.8926–263.7207]	3.3254	[27.3192–34.4365]	98.4574
	Proposed	[39.8926–263.7207]	**3.3950**	[27.2818–34.4322]	**100.4687**
Kendo	DP	[46.8018–325.3662]	5.7321	[33.4782–37.8762]	135.2070
	Proposed	[46.8018–325.3662]	**5.8186**	[33.5635–38.0402]	**136.8867**
Lovebird1	DP	[27.3682–209.7168]	5.1393	[32.0417–40.3639]	102.5171
	Proposed	[27.3682–209.7168]	**5.1852**	[32.0549–40.4131]	**103.6249**

The results listed in Table 4 describe the better performance of our proposed scheme. For example, the BDPSNR gain for “DP” is at least 3.3254 dB for bit-rate range from 39.8926 kbps to 263.7207kbps, and the BDBR gain is at least 98.4574 kbps for PSNR range from 27.3192 dB to 34.4365 dB for test sequence Dancer. Furthermore, the BDPSNR and BDBR gains are increased when the MOBMC is applied for depth reconstruction. The gains of propagation based depth reconstruction are come from bit rate saving, where only 1/8 depth maps are encoded. In other words, the scheme of propagation based depth reconstruction can have better quality results than traditional coding scheme if same bit rate is constrained. Furthermore, the MOBMC can further improve the quality performance of depth reconstruction.

### Summary of the Results

Remote dynamic 3D scene reconstruction is helpful for accurate remote medical processing, and it can be achieved by the combination of 3D scene reconstruction and video streaming. In this combination, the video-rate depth maps are crucial for scene reconstruction, but usually sparse and low resolution depth maps are obtainable. The propagation based depth reconstruction approach has been exploited recently, while it cannot be applied in this application directly since the original color video and accurate motion vectors are not available at the terminal side.

In this paper, we propose to reconstruct video-rate and high definition depth map from the color video stream, and then reconstruct the target dynamic 3D scenes. In this scheme, inaccurate temporal propagation caused by motion vector outliers are detected and processed, propagation hole caused by absent motion vectors are predicted by its neighbors, and dislocation caused by block-wise near-boundary motion vectors are processed by modified overlap block motion compensation. The experimental results shown that our scheme has robust performance for depth map and dynamic 3D scene reconstruction on long distance propagation, even with high compression ratio. Furthermore, our scheme has at least 3.3950 dB quality gains and 100.4687 kbps bandwidth gains when comparing to the benchmark in remote applications.
